# Burden of hospitalizations for pandemic influenza in Slovenia

**DOI:** 10.3325//cmj.2011.52.151

**Published:** 2011-04

**Authors:** Maja Sočan

**Affiliations:** National Institute of Public Health, Ljubljana, Slovenia

## Abstract

**Aim:**

To analyze the 2009/2010 epidemiological data of patients hospitalized for confirmed pandemic influenza in Slovenia.

**Methods:**

We conducted a retrospective analysis of health statistical data collected in an electronic data set Diagnosis-related Group system. Data on age, sex, primary and secondary diagnoses, duration of hospital stay, admission to the intensive care unit, disease outcome, and the week of the admission to the hospital were extracted for patients diagnosed with confirmed influenza virus infection.

**Results:**

A total of 748 (hospitalization rate 37.4/100,000) patients diagnosed with confirmed influenza virus infection were admitted to 19 public hospitals and 7 private acute care providers during the period from September 28, 2009 to April 11, 2010. The highest admission rate was recorded for mid-November 2009. Out of 748 hospitalized patients, 411 (55%) were children younger than 15 years. Influenza was coded as the primary diagnosis in 536 patients. In 35% of the patients, influenza caused viral pneumonia. Fewer than one third of patients (28%) had a pre-existing chronic disease and/or condition predisposing them to complicated or adverse outcomes of influenza, most frequently chronic lung diseases, mainly asthma. A median hospital stay was 2 days for children and 5 days for adult patients. Longer hospitalization was required in patients who had a secondary diagnosis of influenza. Older male individuals suffering from pneumonia and chronic diseases were overrepresented among cases admitted to the intensive care units.

**Conclusions:**

The epidemiological data extracted from the Diagnosis-related Group system in Slovenia were comparable with the data on pandemic patients published elsewhere.

The World Health Organization officially announced the first outbreak of pandemic influenza in the 21st century on June 11, 2009, approximately two months after the first case of infection with pandemic influenza A(H1N1) virus (pH1N109) had been confirmed (1). Enhanced epidemiological and virological surveillance recorded the first pandemic influenza case in Slovenia on June 19, 2009. Infections occurring in the summer months were mostly imported and only rarely spread from patients to other persons. The prevalence of infections peaked in mid-July to decrease slowly by the end of September 2009 (2). The summer emergence of pandemic influenza was not associated with an increased number of visits to primary health care clinics due to acute respiratory infections and influenza-like conditions, suggesting a low number of patients with influenza, particularly those with severe disease (3). During the first, summer wave of pandemic influenza, there were no patients requiring inpatient treatment. In the UK, morbidity caused by pandemic influenza was much higher (4,5).

When the second pandemic wave began in the fall of 2009, it showed characteristics similar to those of seasonal influenza, except that it occurred much earlier than usual. The number of patients seeking medical attention for influenza-like disease began to increase steeply at the end of October and reached its peak in mid-November. There was a parallel increase in the number of hospital admissions, including admissions to the intensive care units (2).

The article provides a descriptive health statistics on patients receiving hospital treatment for confirmed pandemic influenza virus infection in the 2009/2010 season.

## Methods

### Study period and data collection

Data on confirmed hospitalized cases of influenza virus infection were extracted from the electronic Diagnosis-related Groups (DRG) database, run by the National Institute of Public Health (6). The DRG system contains data on acute inpatient treatments and serves as a pricing system for hospital therapy. Data for the DRG system are collected by all Slovenian hospitals that treat acute cases (19 public hospitals and 7 private acute care providers). At discharge, the physician describes individual treatment cases, assigns them to the DRG system using the International Statistical Classification of Diseases and Related Health Problems, 10th revision codes (ICD-10), and identifies the primary diagnosis, ie, the diagnosis that necessitated admission to hospital (7). When this diagnosis is missing, the classification is based on the main symptom, pathological findings, or condition managed in the hospital. Secondary diagnoses refer to diseases and conditions existing before the admission or occurring during inpatient treatment. Secondary diagnosis has a great impact on treatment and/or on whether the patient is likely to experience additional complications. Therapeutic and diagnostic procedures were coded too but were not analyzed.

Data were collected and analyzed for patients with confirmed influenza virus infection coded at discharge as the primary or secondary diagnosis. Using ICD-10 codes, the patients were classified into the following three groups:

- J10.0 influenza with pneumonia; influenza virus confirmed;

- J10.1 influenza associated with respiratory symptoms; influenza virus confirmed;

- J10.8 influenza with other manifestations, such as myocarditis, gastroenteritis, encephalopathy, and others; influenza virus confirmed.

Hospitalized patients identified as ICD-10 code J11 cases (influenza, virus not confirmed) were not included in the analysis.

We supposed that all patients (or at least most of them) who were classified according to ICD-10 as J10.0, J10.1, or J10.8 were laboratory-confirmed cases. Those patients who had influenza-like illness but were not tested or tested negative for pandemic influenza were classified as J11.0, J11.1, or J11.8.

It was nationally recommended to confirm or exclude pandemic influenza in patients with acute respiratory infection that was severe enough to require hospital admission. The laboratory confirmation was also important for cohort isolation of pandemic influenza cases, and microbiological diagnosis determined the antiviral therapy with neuraminidase inhibitors. The testing was done by National Influenza Center and 5 other laboratories using polymerase chain reaction, except in one laboratory that used direct immunofluorescence test.

Data on patient sex, age (years), primary and secondary diagnoses, length of hospital stay, treatment in the intensive care unit, and week (International Standardization Organization week, from Monday 0.00 hours to Sunday 24.00 hours) of admission to the hospital were extracted from the DRG system for the period from the 40th week of 2009 to the 18th week of 2010 (September 28, 2009-April 11, 2010). In Slovenia, no other subtype of influenza A virus than pH1N109 was identified during that period. All hospitalized cases classified as ICD-10 code J10 subgroups were therefore regarded as pandemic influenza cases. Influenza virus B infection was confirmed in the 16th week; confirmed hospitalized cases of influenza were no longer documented during that period (2).

Since the study was designed to retrospectively analyze health statistics data without any personal identifiers, we did not apply for the National Medical Ethics Committee approval.

### Data analysis

Individual categories of categorical variables are presented as percentages and continuous variables as median and range. Differences by sex and age (children under the age of 15 years and adults) were investigated. The prevalence of hospitalization was calculated for the entire population of Slovenia using the population data from December 31, 2009, available on the Web site of the Statistical Office of the Republic of Slovenia (8).

Patients treated in the non-intensive care unit (ICU) hospital wards were compared with those admitted to ICUs according to demographic data, primary/secondary diagnosis, presence of chronic disease that prioritizes patients for vaccination against influenza, presence of pneumonia not caused by influenza virus, and disease outcome. Data on the length of ICU treatment were not available.

We used the χ^2^ test for categorical variables or Mann-Whitney U test for continuous variables that were not normally distributed (9). *P* values of <0.05 were considered significant.

## Results

### Discharge diagnoses

**Figure 1:**
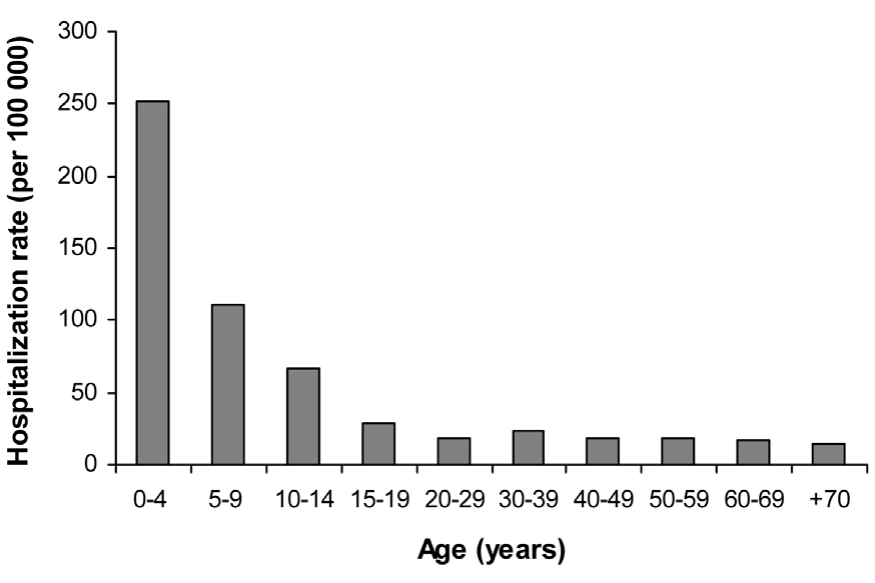
Hospitalization rate (per 100 000 population) for pandemic influenza, by age groups, during the second pandemic wave in Slovenia (from September 28, 2009 to May 11, 2010).

**Figure 2:**
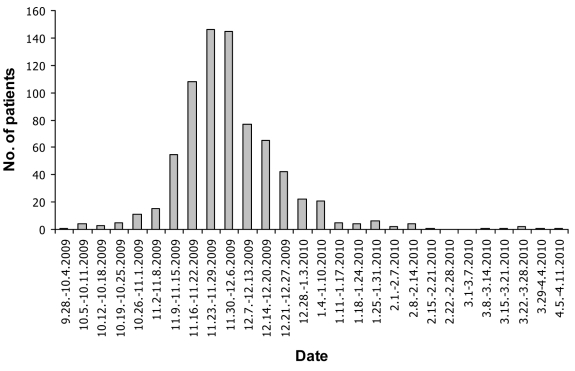
Weekly number of patients admitted with pandemic influenza in Slovenia in 2009/2010 season.

During the 2009/2010 season, 748 patients (401 men – 54% and 347 women – 46%) with the primary or secondary diagnosis of confirmed influenza virus infection (ICD-10: J10.0, J10.1, J10.8) were entered into the DRG system. The hospitalization rate in Slovenia was 37.4 per 100 000 population. The highest hospitalization rate was in the age group 0-4 years (Figure 1). The number of hospital admissions for pandemic influenza peaked in the week from November 23 till November 29, 2009 (Figure 2).

A total of 536 patients (72%) had a primary diagnosis of confirmed influenza virus infection (Table 1). Influenza with concurrent pneumonia (ICD-10 J10.0) was not significantly more frequent in men than in women (37% vs 31%, *P* = 0.502). Influenza associated with pneumonia (as a primary or secondary diagnosis) was established in 24% of children under the age of 15 years and in 47% of adults (*P* < 0.001) (Table 2).

**Table 1:** Number of patients with confirmed influenza virus infection hospitalized in Slovenia in the 2009/2010 season

	No. (%) of patients with influenza with			
Diagnosis-related groups diagnosis	pneumonia	other respiratory symptoms	other manifestations	Total
Primary diagnosis	189 (35)	232 (43)	115 (21)	536 (100)
Secondary diagnosis	68 (32)	109 (51)	35 (17)	212 (100)
Total	257 (34)	341 (46)	150 (20)	748 (100)

**Table 2:** Comparison of demographic data, length of stay, discharge diagnoses, and comorbidities among children (under 15 y of age) and adults hospitalized with pandemic influenza in the 2009/2010 season in Slovenia

	No. (%) or median (range) of		Statistics*
Patient characteristic	children (n = 411)	adults (n = 337),	*P*
Sex-male	218 (53)	183 (54)	0.730
Length of hospital stay (days)	2 (0-33)	5 (0-81)	<0.001
Influenza (primary diagnosis)	309 (75)	227 (67)	0.018
Influenza-associated pneumonia	100 (24)	157 (47)	<0.001
Pneumonia not caused by influenza virus	43 (11)	63 (19)	0.001
Chronic disease or state	53 (13)	158 (47)	<0.001

*χ^2^ test for categorical variables and the Mann-Whitney U test for continuous variables.

Primary diagnoses of 212 patients with influenza as a secondary diagnosis were various, most frequently acute upper or lower respiratory tract infection. Pneumonia was diagnosed in 40, other acute lower respiratory infections in 16, and acute upper respiratory infections in 13 patients. Acute respiratory failure was the primary diagnosis in 27 patients. The primary diagnosis of dehydration was made in 18 patients, mostly in small children, and of febrile convulsions in 8 patients.

### Patients' age structure

The median age of patients with pandemic influenza was 11 years (range 0-88 years). Accurate data on the age of children younger than one year were not available. Men and women did not have significantly different median age (12 vs 10 years, *P* = 0.489). Median age of patients with a primary diagnosis of influenza was lower than that of patients with a secondary diagnosis of influenza (10 vs 17.5 years, *P* = 0.017). A primary diagnosis of influenza was made in 75% of children under the age of 15 years and in 67% of adults (*P* = 0.018).

### Length of hospital stay

**Figure 3:**
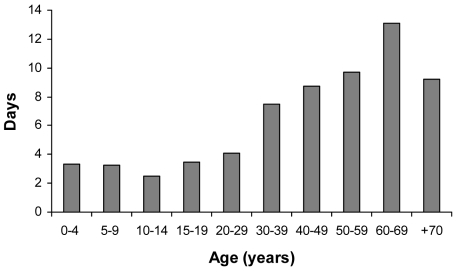
The average length of hospital stay (in days) in patients with pandemic influenza by age groups during the second pandemic wave in Slovenia (from September 28, 2009 to May 11, 2010).

The median length of hospital stay was 3 days (range, 0-81) – 2 days in children (range, 0-33) and 5 days in adults (range, 0-81). On average, children aged 10-14 years had the shortest hospital stay (Figure 3). Older patients with a secondary diagnosis of influenza and patients with a secondary diagnosis of influenza with other coexisting chronic illnesses had longer hospital stay than patients with a primary diagnosis of influenza.

### Secondary diagnoses

In 537 patients (72%), there was no chronic disease or state which could predispose a complicated course of influenza. In the rest of the patients, there was at least one diagnosis of chronic cardiac, lung, metabolic, renal, or liver disorders or diseases compromising the immune system, such as cancer, rheumatoid disorders, congenital or acquired immune deficiency, or other risk factors for complications (pregnancy, obesity). One of these risk factors was identified in 145 patients (19%), 2 in 44 (5.9%), 3 in 20 (2.7%), and 4 in 2 patients (0.2%).

There were no significant differences in chronic illnesses (cardiovascular, pulmonary, liver, kidney, bowel, neurological, autoimmune, or metabolic diseases) and other risk factors (obesity) between male and female patients, with the exception of chronic liver disease (mostly due to alcoholism), which affected more men than women (*P* = 0.004) (Table 3).

**Table 3:** The comparison of chronic diseases or states among 401 male and 347 female patients hospitalized with pandemic influenza in the 2009/2010 season in Slovenia

	No. (%) of patients with chronic disease/state		Statistics*
Chronic disease/ state	male	female	*P*
Cardiovascular disease	38 (9.5)	25 (7.2)	0.265
Respiratory disease	44 (11.0)	44 (13.0)	0.469
Kidney disease	7 (1.7)	5 (1.4)	0.740
Diabetes	16 (3.9)	14 (4.0)	0.975
Bowel disease	5 (1.3)	3 (0.9)	0.612
Liver disease	23 (5.7)	6 (1.7)	0.004
Malignant disease	9 (2.2)	11 (3.2)	0.430
Neurological disease	11 (2.7)	11 (3.2)	0.730
Autoimmune disease	5 (1.3)	8 (2.3)	0.269
Obesity	4 (1.0)	6 (1.7)	0.384

*χ^2^ test for categorical variables.

As expected, significant differences were established between children under the age of 15 years and adults: only 53 children (13%) had a coexisting chronic disease compared with 158 adults (47%, *P* < 0.001) (Table 2). The proportion of patients with several chronic diseases or risk factors for influenza complications was significantly greater in adults (61 of 158 patients; 39%) than in children (5 of 53 patients; 9.4%, *P* < 0.001). The principal comorbidity in children was asthma (25 children).

Pneumonia caused by a bacterium or virus other than influenza virus was diagnosed in 106 patients (14%), with no difference by sex (15% vs 13%, *P* = 0.380). Influenza-associated pneumonia was diagnosed in 11% of children under 15 years of age and in 19% of adult patients (*P* = 0.001). The causative agent of pneumonia remained unidentified in the majority of cases.

### ICU admissions

Fifty-five patients (7%) were treated in the ICU. ICU patients were older, more frequently men, with more comorbidities, and more frequently with influenza-associated pneumonia than non-ICU patients with confirmed pandemic influenza (Table 4).

**Table 4:** Demographic data, diagnosis of influenza (primary/sary), pneumonia, chronic diseases or risk factors for complications and mortality of patients with pandemic influenza treated in intensive care unites (ICU) and non-ICU departments of Slovene hospitals

	No. (%) or median (range) of patients treated in		Statistics*
Patient characteristic	non-ICU (n = 693)	ICU (n = 55)	*P*
Sex-male	364 (53)	37 (67)	0.034
Age (years)	10 (0-87)	62 (3-88)	<0.001
Influenza (primary diagnosis)	513 (74)	32 (42)	<0.001
Influenza-associated pneumonia	218 (31)	39 (71)	<0.001
Pneumonia not caused by influenza virus	88 (13)	18 (33)	<0.001
Absence of chronic illness, risk factors	518 (75)	19 (35)	<0.001
Outcome-death	6 (0.9)	15 (27)	<0.001

*χ^2^ test for categorical variables and the Mann-Whitney U test for continuous variables.

## Discussion

During the second pandemic wave, hospitals in Slovenia admitted 748 patients with confirmed pandemic influenza. The highest admission rate was found among small children (aged 0-4 years) but only a few of them were admitted to the ICU. Among severe cases admitted to the ICU, the majority were older male patients with pre-existing chronic health problems, while one third were without chronic diseases.

A number of countries gathered data on hospitalized patients with confirmed pandemic influenza using recently implemented notification systems and accurate questionnaires comprising demographic, epidemiologic, clinical, and microbiological variables. The results allowed timely updating of guidelines and therapeutic approaches (10-16). Patients admitted with pandemic influenza were in general younger than patients admitted with seasonal influenza in previous seasons. Two thirds of patients with pandemic influenza admitted to the ICU had an underlying chronic condition and were older than those treated in non-ICU departments. Chronic respiratory disease was most common risk factor, followed by cardiovascular diseases (12-14,17).

During the summer pandemic wave, there was enhanced surveillance of the initial 100 influenza cases in Slovenia (3). During the second wave, collecting of individual-level data was discontinued, and only statutory notifications of confirmed pandemic influenza cases were provided. In addition, all Slovene hospitals were asked to supply weekly reports on new admissions for confirmed pandemic influenza cases, which served as a basis to estimate the burden of pH1N109 in hospitals. The number of cases reported weekly by hospitals was lower compared with DRG system (656 vs 748). Two hospitals covering approximately 15% of the population in one Slovenian region did not comply with the demand and they did not submit the data on newly admitted confirmed pandemic cases, which explains the difference. The fall/winter pandemic wave was different than the summer wave and affected all age groups. Epidemiological data for the 2009/2010 fall and winter pandemic wave showed highest influenza rates among school children, followed by older preschool children and under-four-year-olds, with a peak in the 47th week (from November 9-15, 2009) (2). According to the DRG system data, the hospitalization rate for pandemic influenza peaked two weeks later, during the week from November 23-29, 2009. The hospitalization rate for Slovenia (37.4 per 100 000 population) is one of the highest published in the literature (14,17). It may reflect the tendency of Slovene physicians to more readily refer patients to hospital care, particularly small children who are hospitalized for short observation only. This assumption, however, is not based upon evidence. It may be that lower hospitalization rates reported in other studies are due to incomplete notification of cases. While the DRG system covered all hospitalized patients with pandemic influenza in Slovenia, data collection in some other countries, such as the UK, France, and the Netherlands, was based mostly on disease notifications. Although physicians in these countries are statutorily obliged to notify on cases of pandemic influenza, the notifications might not have been consistent: physicians are frequently overworked and therefore forget to keep up with administrative tasks or, more often, do not consider it important to make notification (16).

We found no significant difference between sexes, which is similar to other studies (13-16,18). In contrast to most studies, a Canadian prospective observational study of patients admitted to the ICU with pandemic influenza reported a higher proportion of women (67%) (19).

Children under the age of 15 years accounted for 55% of hospitalized patients although they had markedly lower prevalence of chronic morbidity likely to complicate the course of influenza. On average, children stayed in hospital shorter than adults. A large proportion of pediatric patients was reported by the epidemiological studies that dealt with all admitted patients regardless of disease severity (12,13,20,21), while lower percentages of children and thus older average age of patients was reported by studies focusing on the most serious cases or on patients admitted to the ICU (14,16). In all literature reports, the average and the median ages were markedly lower than those in previous influenza seasons. Moreover, the rate of age-matched hospitalization for Slovenia was lowest for patients aged 70 years and over, suggesting cross-protection against influenza by antibodies present in the elderly (18).

The median hospital stay was 3 days, which is comparable to the results published by other authors. In the UK, the median duration of hospital stay for children was one day longer than in Slovenia, and one day shorter than for adult patients (15). The median hospital stay in our series was equal to that in a Spanish study, which had predominantly more adults and included only non-ICU cases (16). As expected, older patients with underlying chronic diseases and those who had a secondary diagnosis of influenza had longer hospitalization rates.

A diagnosis of at least one chronic illness was made in 28% of patients, which is lower than reported in the Netherlands (55% of non-ICU patients), equal to that reported in Spain (26%), and higher than that reported in France (13-16). Different number of chronic patients reported in these studies was due to different criteria used for hospital admission or inaccurate recording of chronic health problems. In Slovenia inconsistent and inaccurate coding of chronic diseases leads to underestimated rather than overestimated proportion of chronic cases.

Our data showed the highest prevalence of chronic lung diseases, followed by cardiovascular diseases and diabetes. Asthma was the most common chronic lung disease, particularly in children, which is comparable with the findings of other investigators (13-16). Because of the unavailability of data on the prevalence of chronic disorders in the Slovene population, it was not possible to identify chronic diseases associated with an increased risk for admission to hospital during the pandemic season 2009. The diagnosis of obesity was coded in solely 1% of patients and the information on whether those patients had a body mass index exceeding 40 (definition of morbid obesity) was not available. Some studies included a large proportion of severely obese hospitalized individuals, while the figures from the UK study were similar to those in our series (15,16). The differences may be due to the prevalence of obesity in the population or to inconsistent recording of this piece of information at discharge. Also, the DRG system failed to identify the prevalence of smokers among patients with pandemic influenza. In addition, the etiological diagnosis of pneumonia could not be identified, which is one of the limitations of the retrospective health statistics data analysis.

Patients treated in the ICU for confirmed pandemic influenza were for the most part older men with multiple chronic diseases, higher rates of pneumonia, and expected adverse outcomes. There was no discharge diagnosis suggesting a chronic disease for 17 patients (31%) admitted to the ICU. Our results were similar to those of other studies, except that some authors reported a greater number of children requiring treatment in the ICU (22,23).

Retrospective observational studies have a number of limitations inherent to the analysis of health statistics data. The data were anonymized and could therefore not be checked for accuracy. We believe that all patients defined as having the diagnosis of confirmed influenza infection in DGR system were included in our study. The question is, however, how many cases of influenza confirmed during hospital stay were not coded at discharge. Inconsistencies in coding the coexisting chronic diseases at discharge from hospital represent the main limitation of this study. We allow the possibility that children with chronic diseases were underrepresented among our patients.

Analysis of the DRG system data showed that epidemiological characteristics of patients with pandemic influenza in Slovenia were similar to those from other comparable studies. The rate of hospitalization was markedly higher for younger age groups, whereas the disease was most severe in older patients with chronic diseases.

Despite some limitations, health statistics represent a valuable source of information, but one that is too often neglected. Since collecting health statistics takes a lot of time and effort, it is sensible to make use of the information acquired regardless of some inaccuracies or random errors it may contain. Health statistics will serve very little purpose if taken as a data “cemetery.”
